# The Complete mitochondrial genome of *Marmota vancouverensis* (Vancouver Island Marmot)

**DOI:** 10.1080/23802359.2019.1668308

**Published:** 2019-09-20

**Authors:** Zhaonan Hao, Yi Cao

**Affiliations:** Microbiology and Metabolic Engineering of Key Laboratory of Sichuan Province, College of Life Sciences, Sichuan University, Chengdu, China

**Keywords:** *Marmota vancouverensis*, mitochondrial genome, protein-coding genes, phylogenetic

## Abstract

*Marmota vancouverensis*, the only uniquely Canadian marmot, has been listed on the International Union for Conservation of Nature (IUCN) Red List of Threatened Species. The complete mitochondrial genome sequence of *M. vancouverensis* (Vancouver marmot) is presented here firstly, sequenced by next-generation sequencing (NGS). The Vancouver marmot mitogenome is 16,435 bp long, contains 13 protein-coding genes (PCGs), two rRNA genes (12S rRNA and 16S rRNA), 22 transfer RNA (tRNA) genes, and one control region (D-loop). The complete mitochondrial genome sequence provided here could help in study of ecological and evolutionary research of Marmota and conservation genetics of *M. vancouverensis*.

The Vancouver Island marmot is the only one species of marmot only found on Vancouver Island, which is on the top20 list of Threatened Small Mammals of IUCN (Roach 2017). It has been showed that effects of deforestation and habitat fragmentation, the variety of environments (Cardini et al. 2007) and predation pressure, Alle effect (Brashares et al. 2010) are part of population decline (has declinded by 80–90% since the 1980s). To know more about the biological diversity of this species and protect them, more genetic analyses and other detailed morphological studies are needed. Based on this purpose, we assembled the mitochondrial genome of *M. vancouverensis.*

The raw data of whole genome sequencing from Vancouver Island marmot’s liver(Accession no.SAMN09425912, specimen voucher RANI-1049, collected on Vancouver island, British Columbia 49°40′N125°50′W, stored in a freezer in Abbotsford, Canada), provided by Ministry of Agriculture and Lands, Animal Health Center, Abbotsford, British Columbia, Canada, sequenced using HiSeq X Ten, has been used. The raw reads (SRR7403958) were trimmed using Trimmomatic v0.4.0 (Bolger et al. 2014), then assembled and annotated with NOVOPlasty v2.7.2 (Dierckxsens et al. 2016) and MITOS2 (Bernt et al. 2013), respectively. We used *Marmota himalayana* (NC_018367.1) as the reference during assembly. Finally, we get 16,435 bp long, double-stranded circular DNA (GenBank Accession No. MK859897). This mitogenome of *M. vancouverensis* includes 13 protein-coding genes, two ribosomal RNA genes (12S rRNA and 16S rRNA), 22 tRNA genes, and one control region (D-loop) identified by sequence homology (identity 91.24%, coverage 97.8% vs *M. himalayana*; four conserved sequences are totoally identical to *M. himalayana*’s (Chao et al. 2014)) . The contents of A, T, G, and C are 32.24%, 31.76%, 12.58%, and 23.42%. GC contents are 36.00%. All of the PCGs use complete (ATG, ATA) start codon and among them, ten of PCGs have complete stop codon (TAA, TAG, AGA), which also proves the integrity of our assembly. The lengths of 12S rRNA and 16S rRNA genes are 971 and 1,562 bp, respectively. The length of 22 tRNA genes ranges from 59 bp (tRNA-Ser) to 74 bp (tRNA-Leu). The D-loop is 999 bp and lies between the tRNA-Phe and tRNA-Pro.

Phylogenetic analysis of 15 mitogenomes using RAxML v8.2.7(Stamatakis 2014). *Neodon Irene* was used as outgroup. As it turns out that *M. vancouverensis* is colsest to *Marmota himalayana* (Figure 1). The mitogenome of *M. vancouverensis* provides useful resources to study the phylogeny and evolution of marmots, as well as protection.

**Figure 1.**
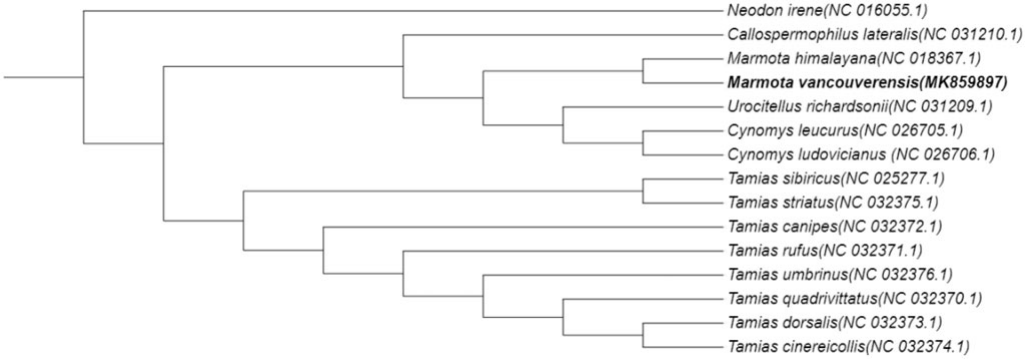
Phylogenetic tree constructed with *M. vancouverensis* and 14 other species mitogenomes. It was constructed based on the alignment of MUSCLE v3.8.425 (Edgar 2004). The bootstrap support values are generated using 100 replications.
